# MV-HAGCN: Prediction of miRNA-Disease Association Based on Multi-View Hybrid Attention Graph Convolutional Network

**DOI:** 10.3390/ijms27083533

**Published:** 2026-04-15

**Authors:** Konglin Xing, Yujing Zhang, Wen Zhu

**Affiliations:** 1 School of Mathematics and Statistics, Hainan Normal University, Haikou 571158, China; 202312070100015@hainnu.edu.cn; 2 Key Laboratory of Data Science and Intelligence Education, Hainan Normal University, Haikou 571158, China; 920092@hainnu.edu.cn; 3 School of Mathematics and Systems Science, Guangdong Polytechnic Normal University, Guangzhou 510665, China

**Keywords:** miRNA-disease association, graph convolutional network, attention mechanism, multi-source data fusion, machine learning

## Abstract

Accurate identification of disease-associated microRNAs (miRNAs) is crucial for elucidating pathogenic mechanisms and advancing therapeutic discovery. Although computational methods, particularly those based on biological networks, have become essential tools for predicting miRNA-disease associations, existing approaches often struggle to comprehensively learn from heterogeneous data and optimize feature representations. To overcome these limitations, we propose the Multi-view Hybrid Attention Graph Convolutional Network (MV-HAGCN). This framework constructs a comprehensive heterogeneous network by integrating multi-source biological information, simultaneously capturing miRNA similarity and disease similarity. We design a hierarchical attention mechanism to enable refined feature learning: first, the Efficient Channel Attention (ECA) module prioritizes information-rich input features, ensuring the model focuses on high-value biological characteristics. Subsequently, the Multi-Head Self-Attention Graph Convolutional Network operates on these refined features. Through iterative message passing and multi-head self-attention, it captures not only direct first-order relationships between nodes but also explicitly models and infers complex, indirect higher-order relationships within the network. This hierarchical design progressively refines feature representations, from channel-level recalibration to global structural dependency modeling, enabling the model to capture both local and high-order relational patterns. Furthermore, a dynamic weight learning strategy adaptively integrates multi-perspective similarity matrices, achieving superior feature complementarity and synergy. Finally, the high-order node representations learned through multi-layer graph convolutions are fed into a multi-layer perceptron for integration and nonlinear transformation, enabling precise prediction of potential miRNA-disease associations. Comprehensive evaluation through five-fold cross-validation on HMDD v2.0 and v3.2 benchmark datasets demonstrates that MV-HAGCN consistently outperforms existing state-of-the-art methods in predictive performance. Case studies targeting key diseases such as breast cancer, lung tumors, and pancreatic disorders revealed that the top 50 miRNAs associated with each of these three conditions were all validated in databases, confirming the practical value of this model in screening candidate miRNAs with high biological relevance.

## 1. Introduction

MicroRNAs (miRNA) represent a family of endogenously expressed non-coding RNAs, typically 18–26 nucleotides long, which are fundamentally involved in essential biological mechanisms such as cellular proliferation, differentiation, and disease development through post-transcriptional regulation of gene expression [1]. Since researchers discovered the close relationship between miRNAs and human diseases in the early twentieth century, this field has become a hotspot in biomedical research [2]. For instance, mutations in let-7 miRNA have been demonstrated to cause abnormal cell division [3], while deletion of the miR-15/16 gene cluster has been implicated in the progression of chronic lymphocytic leukemia [4].

Traditional biological experimental methods, including qRT-PCR [5], Northern blotting [6], and microarray analysis [7], although important for validating specific miRNA-disease associations, suffer from inherent limitations such as long experimental cycles, high economic costs, and limited throughput. With the rapid development of high-throughput sequencing technologies, the number of newly discovered miRNAs has increased dramatically, making it impractical to validate all potential associations through experimental approaches systematically. Therefore, developing efficient and accurate computational prediction methods is of great significance for advancing miRNA functional research and clinical applications [8].

Early computational methods were primarily based on the similarity measure hypothesis, which posits that functionally similar miRNAs are more likely to be involved in phenotypically similar disease processes. Jiang et al. [9] pioneered a prediction system based on hypergeometric distribution scoring to rank candidate miRNAs in heterogeneous human phenome-miRNAome networks. Chen et al. [10] developed the RWRMDA model, which utilizes the random walk with restart algorithm to mine potential associations in miRNA functional similarity networks. Xuan et al. [11] introduced MIDP, which enhanced miRNA functional similarity measurement by weighting family and cluster members more heavily. Despite their effectiveness, similarity-based methods rely heavily on the quality of precomputed similarity measures and often fail to capture indirect relationships that are not explicitly encoded in pairwise similarities [12].

Network propagation methods predict potential associations through information diffusion processes in biological networks. You et al. [13] developed a triple-subgraph heterogeneous network, utilizing depth-first search for path exploration between nodes. Chen et al. [12] proposed the WBSMDA model, which innovatively introduced within-score and between-score metrics to measure similarities between diseases or miRNAs, capable of predicting all miRNA-disease pairs, including isolated nodes. Shi et al. [14] built heterogeneous networks based on known association information and identified co-regulated regions of miRNA-disease from the perspective of protein–protein interaction networks. While network propagation methods improve upon local similarity measures by incorporating global network topology, they typically operate under fixed propagation rules and cannot adaptively learn task-specific patterns [15].

Advances in machine learning have facilitated the continuous development of diverse predictive frameworks. Xu et al. [16] proposed a support vector machine-based classifier incorporating miRNA–target interactions and expression profiles. Chen et al. [17] introduced a random forest model that leverages features constructed from statistical measures, graph-theoretic indices, and matrix factorization. Wang et al. [18] designed the HFHLMDA approach, which utilizes high-order feature representations combined with hypergraph learning. Although traditional machine learning methods have achieved notable success, their performance is often limited by handcrafted features and relatively shallow architectures, which struggle to capture the complex, nonlinear relationships inherent in biological systems [19].

Deep learning methods have demonstrated unique advantages in capturing complex nonlinear features. Chen et al. [20] developed RBMMMDA using a restricted Boltzmann machine, while Wang et al. [21] built a predictor through miRNA-disease association-based functional similarity. Liu et al. [22] extracted feature representations through deep autoencoders and utilized deep random forests for prediction. Despite their expressive power, deep learning models typically assume independent and identically distributed (i.i.d.) inputs, failing to explicitly leverage the graph-structured nature of biological networks [23].

In recent years, graph neural networks have garnered considerable interest owing to their remarkable capability in processing graph-structured information. Li et al. [24] introduced the NIMCGCN framework, which employs graph convolutional operations to derive node embeddings from similarity networks. Tang et al. [25] developed MMGCN, employing GCN encoders to extract features from multiple similarity views and enhancing features through multi-channel attention mechanisms. Li et al. [26] presented HGANMDA, implementing hierarchical graph attention networks to determine the significance of neighboring nodes and various meta-paths.

Building upon these advances, a growing number of GNN-based methods have since been proposed to further enhance predictive performance and address specific challenges. For example, Zhang et al. [27] introduced AGAEMD, integrating graph attention with skip connections and residual encoders, while Peng et al. [28] presented MHCLMDA, which builds multiple KNN-based hypergraphs and learns unified representations via hypergraph contrastive learning. Bi et al. [29] introduced ESGC-MDA based on simple graph convolutional networks with a random message dropping strategy. Ning et al. [30] introduced AMHMDA, which combines multi-view similarity networks equipped with attention mechanisms and hypergraph learning via the introduction of hyper-nodes into a heterogeneous hypergraph structure. Zhong et al. [31] combined graph random propagation networks and attention networks to mitigate over-smoothing issues.

Despite considerable advances in computational methods, existing approaches still face several critical challenges. First, most methods fail to effectively integrate heterogeneous biological information, often relying on single or limited data sources [15], which restricts their ability to capture the complex regulatory mechanisms underlying miRNA–disease associations. Second, traditional graph convolutional networks (GCNs) are prone to over-smoothing as network depth increases [32], leading to diminished discriminative power of node features. Third, current models lack adaptive mechanisms to evaluate the importance of different features [33], making it difficult to prioritize biologically meaningful patterns within complex networks. These limitations highlight the need for a more sophisticated framework that can seamlessly integrate multi-source data, preserve feature distinctiveness through deep architectures, and dynamically weigh feature contributions.

Inspired by the complementary strengths of attention mechanisms in prioritizing informative features [34] and multi-view graph learning in capturing complex relationships [25], we propose the Multi-view Hybrid Attention Graph Convolutional Network (MV-HAGCN) to address these challenges. The main contributions of this work are summarized as follows: (1) A multi-source information fusion strategy for comprehensive graph construction. Unlike existing methods that rely on single or limited data sources, we integrate five distinct miRNA similarities and three disease similarities into a unified heterogeneous graph, providing the model with richer and more complementary biological information—addressing a key limitation in prior works. (2) A novel hierarchical attention architecture for refined feature learning. Building upon this multi-source foundation, we propose a dual-stage attention mechanism that goes beyond simple feature aggregation. First, an Efficient Channel Attention (ECA) module preprocesses input features to prioritize high-value biological characteristics. Subsequently, a Multi-Head Self-Attention Graph Convolutional Network explicitly models complex, higher-order relationships, mitigating the over-smoothing issue common in deep graph networks. (3) A dynamic weight learning strategy for adaptive feature fusion. To optimally combine information from multiple views and network depths, we further introduce an adaptive mechanism that learns to assign importance coefficients to different similarity matrices and GCN layers. This ensures that the model can dynamically balance contributions from various sources, achieving superior feature synergy.

Through systematic experimental validation, MV-HAGCN consistently achieves superior performance across multiple evaluation metrics. Compared with existing state-of-the-art methods, our model demonstrates distinct advantages in feature learning and relational modeling. Case studies further confirm the practical utility of the model across diverse disease contexts, establishing it as a reliable computational tool for identifying miRNA-related biomarkers.

## 2. Results

In this work, we employed a hierarchical attention-based graph convolutional network (GCN) to learn embeddings for miRNA–disease associations (MDAs) from a heterogeneous network. The GCN comprised two layers (L=2), each with an embedding size of 256. After feature combination and reconstruction, the final dimensionality of the miRNA–disease pair (MDP) representations was set to 256. The model was trained for 2000 epochs with a learning rate of 0.0005 and weight decay of 0.0005.

To evaluate the predictive performance of MV-HAGCN, we adopted standard evaluation protocols [35] and assessed the model using Accuracy (ACC), Area Under the ROC Curve (AUC), and Area Under the Precision-Recall Curve (AUPR). AUC reflects the model’s overall ranking ability, ACC measures the proportion of correctly predicted associations, and AUPR is particularly informative for imbalanced datasets.

Additionally, we employed both 5-fold cross-validation schemes to systematically examine the robustness and generalization capacity of the MV-HAGCN framework.

### 2.1. Comparison with Baseline Methods

**Figure 1:**
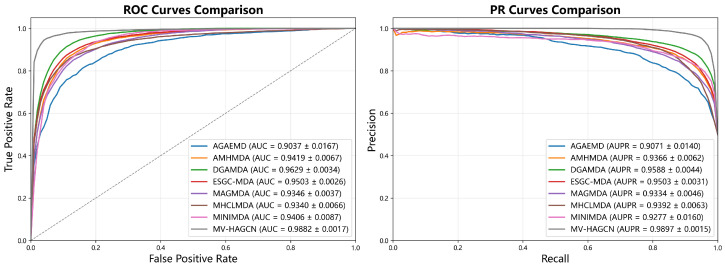
Comparison results of MV-HAGCN and baseline methods under 5-fold CV on HMDD v2.0.

**Figure 2:**
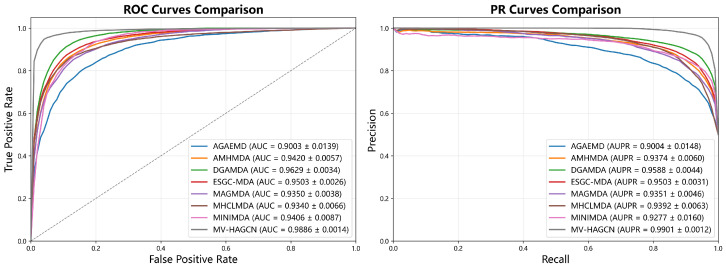
Comparison results of MV-HAGCN and baseline methods under 5-fold CV on HMDD v3.2.

To demonstrate the superior performance of MV-HAGCN, we compare it with six state-of-the-art (SOTA) baseline methods, including AGAEMD (2023) [27], AMHMDA (2023) [30], DGAMDA (2022) [36], MHCLMDA (2022) [28], MINIMDA (2022) [37], ESGC-MDA (2025) [29] and MAGMDA (2026) [38]. For a standardized comparison, each model was implemented using the hyperparameters reported in its original publication. The comparative results are presented in Figure 1 and Figure 2. On HMDD v2.0, MV-HAGCN attains an AUC of 0.9882 and AUPR of 0.9897, outperforming the best baseline model DGAMDA by 2.53% in AUC and 3.09% in AUPR. On the larger HMDD v3.2 dataset, MV-HAGCN achieves an AUC of 0.9886 and AUPR of 0.9901, surpassing DGAMDA by 2.57% in AUC and 3.13% in AUPR. Notably, the gain is most substantial in AUPR, which is particularly important for imbalanced datasets, indicating that MV-HAGCN excels at identifying true positive associations while maintaining low false positive rates. For additional evaluation metrics, please refer to Table S6 in the Supplementary Materials.

### 2.2. Parameter Sensitivity

To identify the optimal configuration of MV-HAGCN and assess the impact of key hyperparameters on predictive performance, we systematically investigated the effects of learning rate, embedding dimension, and network depth. The results of these analyses informed our final model selection and provided insights into the behavior of the model under varying settings.

#### 2.2.1. Learning Rate Selection

**Figure 3:**
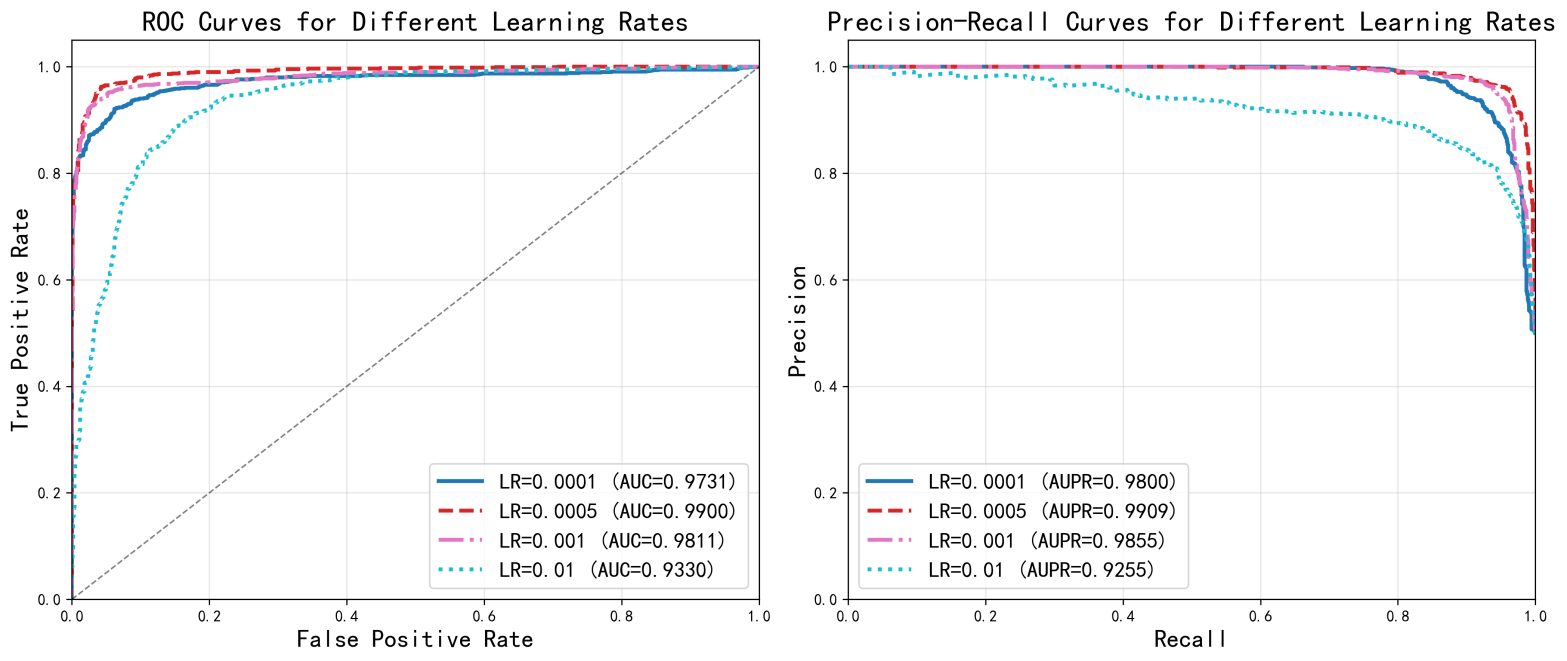
Analysis of different learning rates.

The learning rate governs the step size in gradient-based optimization, directly influencing both training stability and convergence speed. We evaluated four learning rates ranging from 0.0001 to 0.01, with results shown in Figure 3. The model achieved optimal performance at a learning rate of 0.0005, attaining AUC and AUPR values of 0.9900 and 0.9909, respectively. A lower learning rate (0.0001) resulted in slow convergence, whereas higher rates (0.001–0.01) led to training instability and marked performance degradation. These findings underscore the importance of selecting a moderate learning rate to balance convergence efficiency and model robustness.

#### 2.2.2. Embedding Dimension Determination

**Figure 4:**
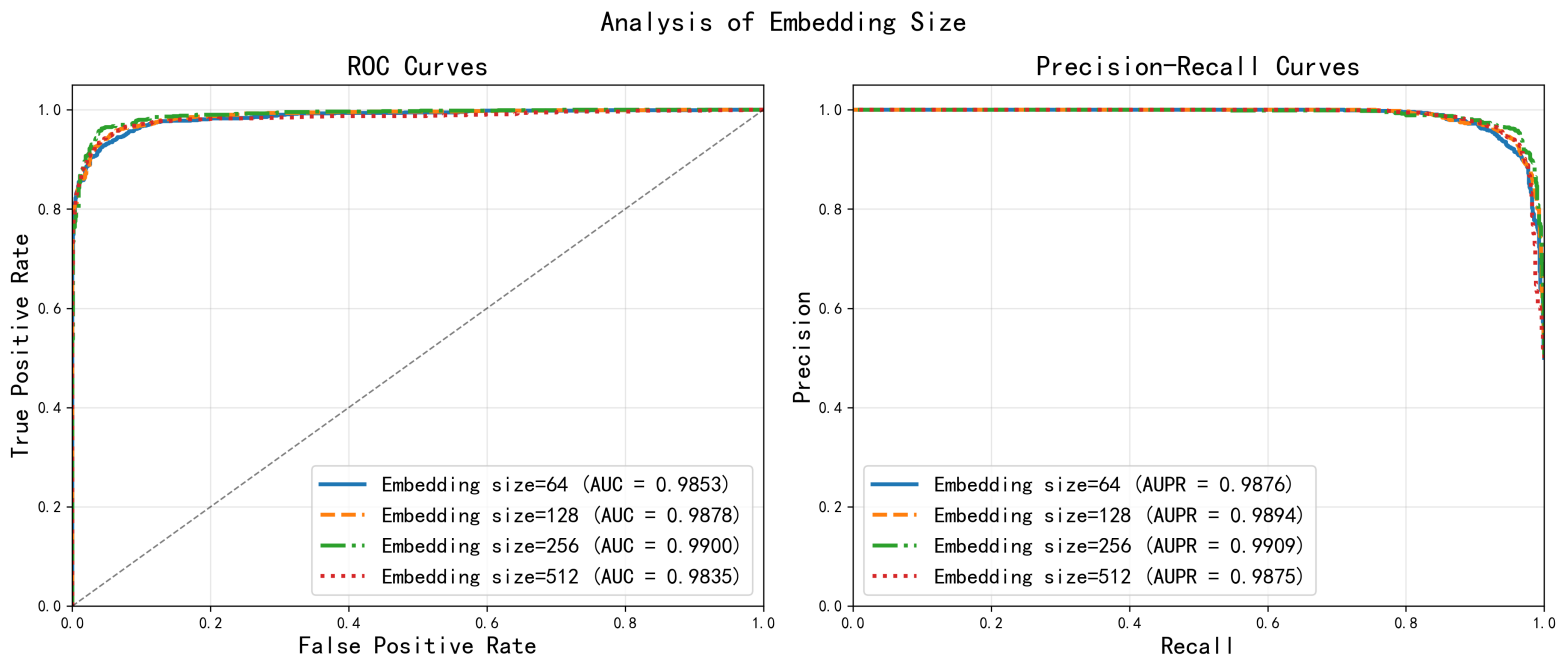
Analysis of different embedding sizes.

The embedding dimension determines the capacity of node representations to encode latent features. We tested the dimensions of 64, 128, 256 and 512, as illustrated in Figure 4. The 256 dimensional embedding yielded the highest AUC (0.9900), achieving an optimal trade-off between representational power and generalization. Lower dimensions (64 and 128) offered insufficient capacity to capture complex relationships, while the 512 dimensional setting led to overfitting, as evidenced by degraded validation performance. This suggests that a moderate embedding size is essential for modeling underlying structures without memorizing noise.

#### 2.2.3. Network Depth Optimization

**Figure 5:**
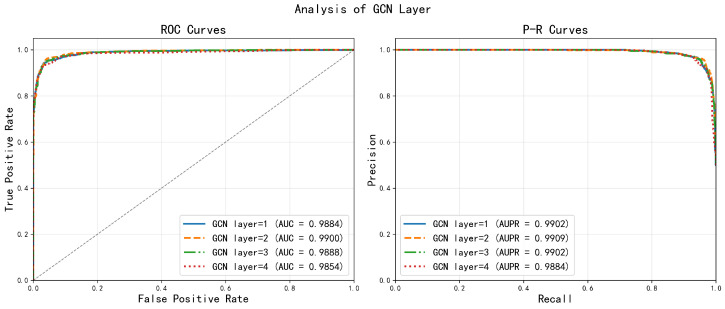
Analysis of different GCN layers.

The depth of the network dictates the scope of information propagation within graph convolutional layers. We compared architectures with one to four layers (Figure 5). The two-layer configuration delivered the best performance (AUC = 0.9900). A single layer exhibited a limited receptive field and failed to capture higher-order dependencies, while deeper networks (three or four layers) suffered from over-smoothing, where node features become indistinguishable, thereby reducing discriminative power. Thus, two layers provide an optimal balance between incorporating multi-hop information and maintaining feature distinctiveness.

#### 2.2.4. Hyperparameters Evolution Analysis

**Figure 6:**
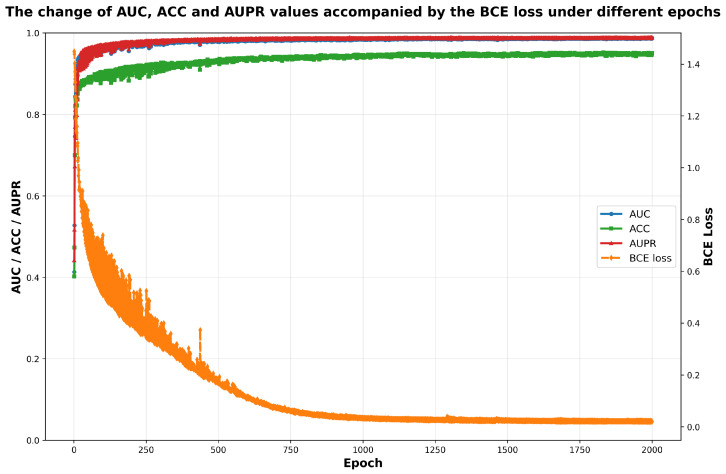
The trends of AUC, ACC, and AUPR alongside BCE loss across training epochs.

**Figure 7:**
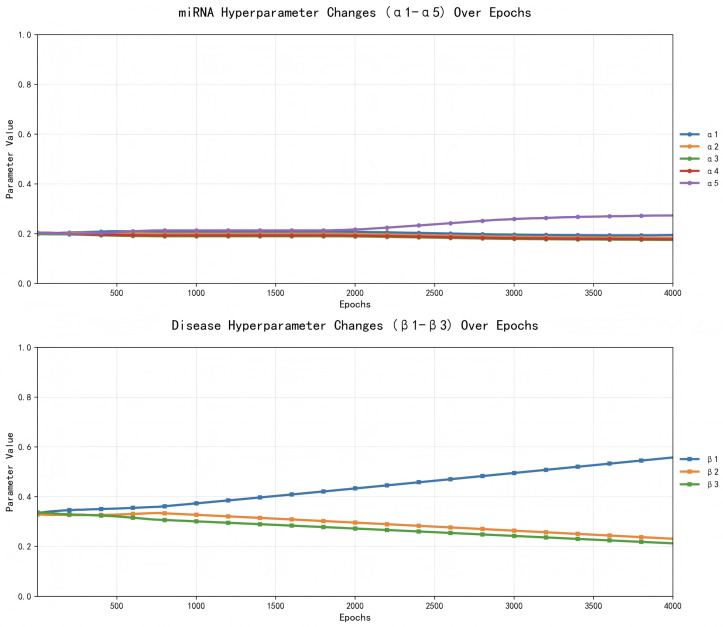
The evolution of hyperparameters across training epochs.

In addition to fixed hyperparameters, MV-HAGCN learns adaptive fusion weights for similarity matrices ($\alpha_{1}$–$\alpha_{5}$ control the contributions of five miRNA similarities: functional, Gaussian kernel, lncRNA-based, sequence and family; $\beta_{1}$–$\beta_{3}$ control the contributions of three disease similarities: semantic, Gaussian kernel, and lncRNA-based). As shown in Figure 6 and Figure 7, after approximately 1600 training epochs, the binary cross-entropy loss stabilizes, and the learned weights converge. The final weight distribution reveals that miRNA family similarity ($\alpha_{5}$) and disease Gaussian kernel similarity ($\beta_{2}$) receive the highest contributions, indicating that functional characteristics are particularly informative for association prediction. This adaptive mechanism enables the model to automatically prioritize the most relevant data sources.

Based on systematic analysis, the optimal configuration for the model is determined as follows: learning rate of 0.0005, embedding dimension of 256, and network depth of 2 layers. This setup maximizes predictive accuracy while effectively mitigating overfitting and over-smoothing.

### 2.3. Ablation Experiments Analysis

**Figure 8:**
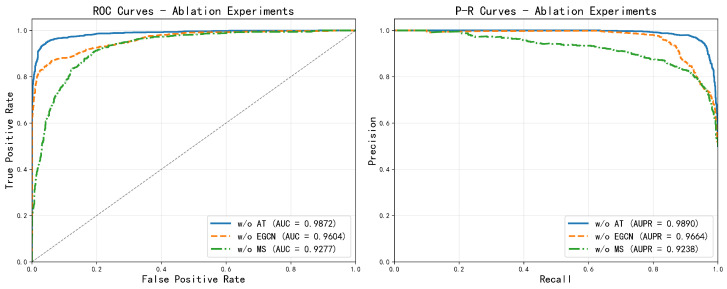
Analysis of ablation experiments. (w/o AT): removing the attention mechanism, (w/o EGCN): removing the enhanced graph convolution, (w/o MS): removing the multi-source fusion.

To rigorously evaluate the necessity of each core component in MV-HAGCN, we formulate the hypothesis that all three modules—namely the attention mechanism, the enhanced graph convolution, and the multi-source information fusion—are hypothesized to contribute positively to the overall predictive performance, and that their removal will lead to measurable performance degradation. Based on this hypothesis, we conducted systematic ablation experiments by establishing three variants: removing the attention mechanism (w/o AT), removing the enhanced graph convolution (w/o EGCN), and removing multi-source fusion (w/o MS). As shown in Figure 8.

The experimental findings confirm that the absence of the attention mechanism causes the AUC to decline to 0.9872, indicating that this module effectively enhances the representational capacity of node features. Removal of the enhanced graph convolution leads to more significant performance degradation, with AUC decreasing to 0.9604, verifying the crucial role of this module in capturing complex graph structural information. The most substantial impact arises from the absence of multi-source information fusion, where AUC sharply declines to 0.9277, emphasizing the importance of integrating multi-source similarity data for biological association prediction.

All three core modules contribute substantially to improving model performance. Among them, the multi-source information fusion module contributes the most, followed by the enhanced graph convolution, while the attention mechanism also demonstrates stable performance gains. This finding confirms the rationality of the model architecture design, where all components work synergistically to ensure the final prediction accuracy. Notably, the removal of multi-source fusion causes the most severe performance drop, far exceeding the degradation caused by removing the enhanced graph convolution or the attention mechanism. This substantial decline underscores that the integration of multiple complementary similarity measures is not merely additive but rather provides essential biological context. In miRNA-disease association prediction, a single similarity modality often captures only a limited aspect of the underlying regulatory mechanisms. For instance, sequence similarity reflects evolutionary conservation, while functional similarity captures pathway-level co-regulation. Removing multi-source fusion forces the model to rely on a less complete representation, thereby losing the synergistic interplay among these diverse biological perspectives. This result aligns with the biological intuition that miRNA-disease interactions are shaped by multifaceted factors, and that a holistic view incorporating multiple data types is necessary for accurate prediction.

### 2.4. Case Studies

To evaluate the model’s potential for clinical deployment, we conducted comprehensive analyses on five clinically significant disease types: esophageal neoplasms, breast neoplasms, lymphoma, lung neoplasms, and pancreatic neoplasms. A rigorous disease-wise hold-out validation strategy was implemented to minimize information leakage. Specifically, for each target disease, we removed all known associations involving that disease from the training set and retrained MV-HAGCN from scratch using the remaining associations. The validation procedure consisted of three steps. First, known miRNA–disease associations were partitioned into training and test sets, with the test set containing all associations for the target disease and the training set comprising the remaining associations. Next, the trained model was applied to compute association probabilities between all miRNAs and the target disease, and the top 50 candidate miRNAs with the highest prediction scores were selected. The full lists of the top 50 predicted miRNAs for each disease are provided in Supplementary Tables S1–S5 and the quantitative evaluation metrics are summarized in Supplementary Table S7. Finally, systematic validation of prediction results was performed through manual retrieval of two major specialized databases: HMDD v4.0 [39] and dbDEMC [40], where database-confirmed associations are indicated with “+” and unconfirmed ones with “-”.

**Figure 9:**
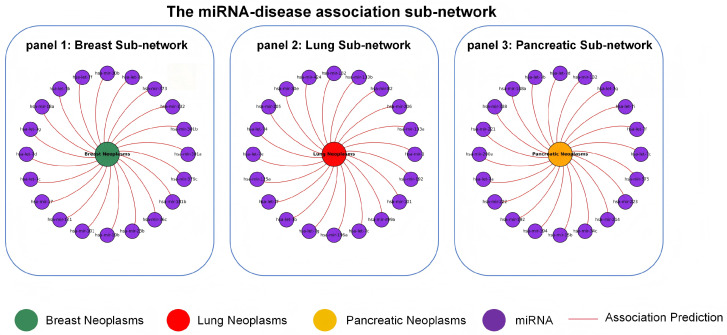
The miRNA-disease association sub-network. The figure presents three sub-networks illustrating the top 20 predicted miRNAs associated with breast neoplasms (green), lung neoplasms (red), and pancreatic neoplasms (orange). Purple circular nodes represent miRNAs, and red edges indicate predicted miRNA-disease associations identified by MV-HAGCN. Due to space limitations, only the top 20 predicted miRNAs are shown for each disease; the complete lists of the top 50 predictions are available in Supplementary Tables S1–S5 and the quantitative evaluation metrics are summarized in Supplementary Table S7.

Breast neoplasms are among the most prevalent malignancies in women worldwide and exhibit strong correlations between molecular subtypes and miRNA expression patterns. Accumulating evidence indicates that miRNAs act as master regulators of breast neoplasm initiation and progression, highlighting their substantial diagnostic and therapeutic potential [41]. In this study, we applied the MV-HAGCN model to predict miRNAs associated with breast neoplasms. The prediction results are presented in Panel 1 of Figure 9. Notably, all top 50 predicted miRNAs were confirmed in the databases (HMDD v4.0 and dbDEMC). Among these, hsa-mir-301a and hsa-mir-301b were identified as key predictive factors and are annotated in dbDEMC as important regulators of breast neoplasms, where they promote cancer progression by modulating immune cell infiltration and angiogenesis within the tumor microenvironment. Similarly, hsa-let-7a—a highly conserved member of the let-7 family—is documented in HMDD v4.0 to suppress breast neoplasm cell proliferation and metastasis by downregulating oncogenes such as RAS and HMGA2 [42]. Another significant predictor, hsa-mir-132, has been shown to influence metabolic reprogramming in breast neoplasm cells through epigenetic mechanisms, particularly by targeting the SIRT1/PGC-1α pathway to modulate mitochondrial function. A prospective time-course simulation using sequential HMDD versions was also conducted for breast neoplasms, and the results are presented in Supplementary Table S9.

Lung neoplasms represent one of the most prevalent and lethal malignancies worldwide, underscoring the critical need for early diagnostic biomarkers. MicroRNAs (miRNAs) function as key regulators in the initiation, progression, and metastasis of lung neoplasms, positioning them as promising diagnostic and therapeutic targets [43]. In this study, the MV-HAGCN framework was employed to predict lung neoplasm-associated miRNAs. The complete prediction results are presented in Panel 2 of Figure 9. Notably, all top 50 predicted miRNAs were confirmed in specialized databases, including HMDD v4.0 and dbDEMC, achieving a 100% confirmation rate in the databases. Specifically, hsa-mir-1, which ranked first in the prediction list, is documented in both HMDD v4.0 and dbDEMC to modulate lung neoplasm progression by regulating multiple target genes, including inhibiting cell proliferation and promoting apoptosis [44]. Additionally, multiple members of the let-7 family, such as hsa-let-7d and hsa-let-7e, were significantly enriched in the prediction results, consistent with database records of this family as tumor suppressors in lung neoplasms. The let-7 family inhibits tumor growth by regulating oncogene expression, including RAS and HMGA2 [45]. Furthermore, hsa-mir-205 has been identified as a tissue-specific biomarker with substantial clinical utility for diagnosing lung squamous cell carcinoma, and its expression levels correlate strongly with tumor staging and patient prognosis [46].

Pancreatic neoplasms are among the most lethal malignancies worldwide, with a five-year survival rate below 10% for pancreatic ductal adenocarcinoma. Elucidating their molecular mechanisms is critical for advancing early diagnosis and targeted therapies. Accumulating evidence indicates that microRNAs (miRNAs) play pivotal roles in the initiation, progression, metastasis, and chemoresistance of pancreatic neoplasms, positioning them as promising biomarkers and therapeutic targets [47]. In this study, we systematically predicted miRNAs associated with pancreatic neoplasms using the MV-HAGCN framework. The results show that all top-50 candidate miRNAs ranked by prediction scores were confirmed in the specialized databases HMDD v4.0 and dbDEMC, achieving a 100% confirmation rate in the databases, as illustrated in Panel C of Figure 9. Specifically, hsa-let-7c was among the top-ranked predictions and is documented in dbDEMC to inhibit multiple key oncogenes, including KRAS and MYC, thereby blocking cell cycle progression and suppressing proliferation of pancreatic neoplasm cells [48]. As another key biomarker, hsa-mir-221 is validated in HMDD v4.0 to promote pancreatic neoplasm progression by suppressing PTEN-regulated AKT signaling [49]. Furthermore, hsa-mir-132 influences energy metabolism in cancer cells through regulating metabolic reprogramming, whereas hsa-mir-375, a tissue-specific miRNA, demonstrates diagnostic potential for pancreatic neoplasms in serum. Together, these findings provide deeper insights into the pathogenesis of pancreatic neoplasms.

These case analyses provide evidence for the effectiveness of MV-HAGCN in identifying disease-associated miRNAs. Systematic validation using two major specialized databases, HMDD v4.0 and dbDEMC, offers robust biological support for the predicted associations. While certain predictions necessitate further experimental verification, they nonetheless present promising directions for elucidating the functional roles of miRNAs in disease pathogenesis and progression. Ultimately, these findings may facilitate the discovery of diagnostic biomarkers and therapeutic targets for relevant diseases.

## 3. Discussion

In this study, we proposed MV-HAGCN, a multi-view hybrid attention graph convolutional network for miRNA-disease association prediction. Experimental results on HMDD v2.0 and v3.2 demonstrate that MV-HAGCN consistently outperforms existing state-of-the-art methods across AUC, AUPR. Beyond these quantitative improvements, we next examine the architectural factors contributing to this performance gain, interpret the biological relevance of the predictions, and discuss the practical limitations of the current framework.

The superior performance of MV-HAGCN can be attributed to three key innovations. First, the multi-source fusion module integrates five complementary miRNA similarities and three disease similarities, allowing the model to leverage heterogeneous biological information that single-view methods cannot access. Notably, the learned fusion weights (Figure 7) assign the highest importance to miRNA family similarity ($\alpha_{5}$) and disease Gaussian kernel similarity ($\beta_{2}$). This indicates that evolutionary conservation (family membership) and topological proximity in known association networks (GIP) are the most discriminative features for predicting novel associations—a finding that underscores the value of integrating both intrinsic sequence-based properties and network-derived relationships. Second, the hierarchical attention mechanism addresses the over-smoothing problem inherent in deep graph convolutional networks. By applying Efficient Channel Attention (ECA) prior to graph convolution and incorporating multi-head self-attention within the convolutional layers, the model preserves feature distinctiveness even at depth two (Figure 5). This enables simultaneous capture of first-order local neighborhood information and higher-order dependencies without homogenizing node representations. Third, the adaptive weight fusion strategy dynamically balances outputs from shallow and deep layers, mitigating the risk of over-amplifying noise from deeper layers—a common failure mode in traditional GCNs. Together, these innovations enable MV-HAGCN to achieve robust performance without requiring excessive network depth.

Beyond architectural considerations, the predictions generated by MV-HAGCN exhibit meaningful biological patterns. The case studies validated the top 50 predicted miRNAs against HMDD v4.0 and dbDEMC, achieving a 100% validation rate. However, rather than merely confirming predictive accuracy, we further investigate why these particular miRNAs were ranked highly. For instance, the consistent enrichment of the let-7 family (hsa-let-7a, hsa-let-7c, hsa-let-7d) across all three cancers can be explained by their high miRNA family similarity ($\alpha_{5}$) scores, as miRNAs within the same family often share seed regions and target similar oncogenic pathways. Specifically, the let-7 family has been well characterized for its role in cell differentiation and cancer suppression [42], with direct regulation of oncogenes such as RAS [45] and involvement in pancreatic cancer progression [48]. This suggests that MV-HAGCN effectively leverages family-level functional coherence to prioritize biologically relevant candidates. Similarly, hsa-mir-301a and hsa-mir-301b, which ranked highly for breast cancer, exhibit strong Gaussian kernel similarity ($\alpha_{2}$) due to their shared association profiles with immune-related diseases, reflecting their known roles in modulating immune infiltration and angiogenesis [50]. In the case of pancreatic cancer, hsa-mir-221 was prioritized largely due to its high GIP similarity with other miRNAs involved in the PTEN/AKT pathway [49]. These examples illustrate that the model’s rankings are not arbitrary but are grounded in quantifiable similarity features—family membership, network proximity, and functional consistency—that align with established molecular mechanisms. This interpretability enhances the model’s utility as a hypothesis-generation tool for experimental validation.

While MV-HAGCN achieves state-of-the-art predictive performance, several limitations warrant discussion. First, the quadratic complexity $O({\left(m+n\right)}^{2})$ of graph convolution operations raises scalability concerns for larger biomedical networks [51]. For the HMDD v3.2 dataset (N≈1450 nodes), the training time of MV-HAGCN is approximately 1291 s on an NVIDIA GeForce RTX 3050 Ti Laptop GPU, representing a moderate level of computational efficiency among the compared methods (detailed runtime comparisons are provided in Supplementary Table S8). For networks exceeding 5000 nodes, however, graph sampling or sparse attention mechanisms may be necessary to maintain practical training times [52]. Second, the validation of predicted associations relies heavily on database evidence (HMDD v4.0 and dbDEMC), which introduces potential confirmation bias [53]. Notably, HMDD v4.0 was used for post-hoc validation in case studies, while training was conducted on HMDD v3.2. Quantitative analysis reveals that HMDD v3.2 contains 12,446 associations, and HMDD v4.0 contains 53,530 associations.Quantitative analysis reveals that nearly one-quarter of the associations in HMDD v4.0 originate from the training set, indicating partial overlap between the two databases.Future work should incorporate experimentally derived associations from independent sources or employ prospective validation to more rigorously assess biological novelty. Third, the current framework treats miRNA-disease associations as binary links, omitting regulatory direction and dynamic expression patterns. Integrating multi-omics data such as single-cell sequencing or temporal expression profiles could enable finer-grained predictions [54]. Finally, extending the framework to incorporate spatiotemporal heterogeneity or multimodal data (e.g., methylation, copy number variation) represents a promising avenue for capturing the full complexity of miRNA-mediated regulation [55].

## 4. Materials and Methods

### 4.1. Datasets

The experimental data used in this study were obtained from HMDD v2.0 [56] and HMDD v3.2 [57]. For instance, HMDD v3.2 comprises a more comprehensive set of associations between miRNAs and diseases. From this database, we curated 853 miRNAs, 591 diseases, and 12,446 experimentally validated associations. Within the MV-HAGCN framework, we constructed an association network represented by a binary matrix denoted as $A=\left[a_{ij}\right]\in R^{N_{m}\times N_{D}}$, where $N_{m}$ and $N_{D}$ represent the numbers of miRNAs and diseases, respectively. Each entry $a_{ij}$ is set to 1 if miRNA $m_{i}$ is known to be associated with disease $d_{j}$, and 0 otherwise.

**Figure 10:**
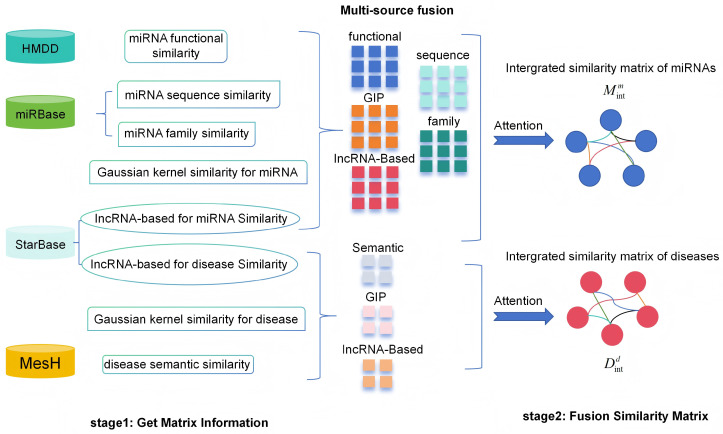
The overall workflow of multi-source fusion. First, multiple similarity measures for miRNAs and diseases are extracted from various databases such as HMDD, miRBase, StarBase, and MeSH. For miRNAs, these include functional similarity, sequence similarity, family similarity, Gaussian kernel similarity, and lncRNA-based similarity. For diseases, they include semantic similarity, Gaussian kernel similarity, and lncRNA-based similarity. These measures are constructed as initial similarity matrices (stage 1). Subsequently, an attention mechanism is applied to adaptively weight and fuse each similarity matrix, generating an integrated miRNA similarity matrix and an integrated disease similarity matrix (stage 2). Finally, these two fused matrices are combined with the known miRNA–disease association matrix to construct a heterogeneous network for subsequent graph convolutional networks, providing the model with comprehensive and complementary initial features.

We computed miRNA-miRNA similarities and disease-disease similarities from multiple perspectives, as shown in Figure 10, including miRNA functional similarity, miRNA sequence similarity, miRNA family similarity, lncRNA-based miRNA similarity, disease semantic similarity, lncRNA-based disease similarity, and Gaussian Interaction Profile (GIP) kernel similarity for both miRNAs and diseases. Detailed descriptions of each similarity measure are provided below. Detailed descriptions of each similarity measure are provided as follows:

#### 4.1.1. miRNA Functional Similarity

We retrieved the miRNA functional similarity scores from the HMDD database [57]. These scores are arranged in a square matrix $F_{m}\in R^{N_{m}\times N_{m}}$, where $N_{m}$ denotes the total number of miRNAs. Each entry $F_{m}\left(m_{i},m_{j}\right)$ quantifies the functional similarity between miRNAs $m_{i}$ and $m_{j}$, with larger values reflecting closer functional relationships.

#### 4.1.2. miRNA Sequence Similarity

Motivated by the observation that miRNAs sharing sequence similarities often target common genes or participate in related biological pathways, we incorporated sequence information to compute miRNA similarity. The miRNA sequences were obtained from the miRBase database [58]. The sequence similarity between a pair of miRNAs, $m_{i}$ and $m_{j}$, was subsequently calculated using the Levenshtein distance [59], as follows:

(1) $$S_{\mathrm{seq}}\left(m_{i},m_{j}\right)=1-\frac{\mathrm{distance}(m_{i},m_{j})}{\max(\mathrm{len}\left(m_{i}\right),\mathrm{len}\left(m_{j}\right))}$$

#### 4.1.3. miRNA Family Similarity

Drawing on the observation that miRNAs within the same family often exhibit similar disease associations, we integrated family information from miRBase into the similarity matrix. Specifically, the family similarity matrix $S_{\mathrm{fam}}$ is defined such that it assigns a value of 1 to any pair of miRNAs $m_{i}$ and $m_{j}$ that belong to the same family, as follows:

(2) $$S_{\mathrm{fam}}\left(m_{i},m_{j}\right)=\left\{\begin{array}{cc} 1, & \mathrm{if}\;m_{i}\;\mathrm{and}\;m_{j}\;\mathrm{belong}\;\mathrm{to}\;\mathrm{the}\;\mathrm{same}\;\mathrm{family}\\ 0, & \mathrm{otherwise} \end{array}\right.$$

#### 4.1.4. Disease Semantic Similarity

The semantic similarity between diseases was derived from their Medical Subject Headings (MeSH) terminology [60] and corresponding Directed Acyclic Graphs (DAGs). We quantify the semantic influence of disease *d* on disease *D* through the following recursive definition:

(3) C1(d,D)=1,ifd=Dmaxμ·C1(d′,D)γ·C1(d′,D)∣d′∈children(d),otherwise

where *d* represents any ancestor node of *D* in the DAG hierarchy, and μ denotes the semantic decay coefficient (default value: 0.5). The cumulative semantic value for disease *D* is then obtained by:

(4) $$V_{1}\left(D\right)=\sum_{d\in A(D)}C_{1}\left(d,D\right)$$

with A(D) defining the complete ancestor set of *D*, including *D* itself. The semantic similarity between two diseases $d_{i}$ and $d_{j}$ is consequently computed as follows:

(5) $$S_{1}\left(d_{i},d_{j}\right)=\frac{\sum_{d\in A\left(d_{i}\right)\cap A\left(d_{j}\right)}\left[C_{1}\left(d,d_{i}\right)+C_{1}\left(d,d_{j}\right)\right]}{V_{1}\left(d_{i}\right)+V_{1}\left(d_{j}\right)}$$

While the above measure effectively captures hierarchical relationships within DAGs, it assumes that all ancestor terms contribute equally to the semantic value of a disease. However, in practice, disease-specific terms that appear in only a few DAGs often carry more discriminative information than common terms shared across many diseases. To address this limitation, we introduce an enhanced contribution metric that accounts for the specificity of disease terms [11]. For a disease *d* unique to the DAG of *D*, its semantic significance is weighted as follows:

(6) $$C_{2}\left(d,D\right)=-\log\left(\frac{{\mathrm{count}}_{\mathrm{DAGs}}\left(d\right)}{N_{\mathrm{diseases}}}\right)$$

where ${\mathrm{count}}_{\mathrm{DAGs}}\left(d\right)$ indicates the frequency of *d* across all disease DAGs, and $N_{\mathrm{diseases}}$ is the total disease count. Following the analogous computational framework, we define:

(7) $$V_{2}\left(D\right)=\sum_{d\in A(D)}C_{2}\left(d,D\right)$$

(8) $$S_{2}\left(d_{i},d_{j}\right)=\frac{\sum_{d\in A\left(d_{i}\right)\cap A\left(d_{j}\right)}\left[C_{2}\left(d,d_{i}\right)+C_{2}\left(d,d_{j}\right)\right]}{V_{2}\left(d_{i}\right)+V_{2}\left(d_{j}\right)}$$

Thus, $S_{1}$ emphasizes the structural topology of DAGs, capturing the shared hierarchical ancestry between diseases, whereas $S_{2}$ assigns higher weights to terms that are specific to a disease, reflecting its unique semantic characteristics. By combining these two complementary measures, we obtain a more comprehensive representation of disease semantics. The two semantic similarity measures are integrated to form a composite metric. The final disease semantic similarity between $d_{i}$ and $d_{j}$ is computed as the average of the two aforementioned scores:

(9) $$S\left(d_{i},d_{j}\right)=\frac{S_{1}\left(d_{i},d_{j}\right)+S_{2}\left(d_{i},d_{j}\right)}{2}$$

The resulting matrix *S* constitutes the final disease semantic similarity matrix.

#### 4.1.5. Gaussian Kernel Similarity

Following established methodologies [61], MV-HAGCN computes the Gaussian Interaction Profile (GIP) kernel similarity, a widely adopted technique in biological network analysis [62]. The computation relies on the known miRNA–disease association matrix *A*. For a pair of miRNAs $m_{i}$ and $m_{j}$, their interaction profiles are represented by the corresponding row vectors $R_{i}$ and $R_{j}$ from *A* [63]. The GIP-based similarity between them is defined as follows:

(10) $$K_{m}\left(m_{i},m_{j}\right)=\exp\left(-\lambda_{m}{∥R_{i}-R_{j}∥}^{2}\right)$$

where $\lambda_{m}$ is a bandwidth parameter controlling the kernel scale, given by:

(11) $$\lambda_{m}=\lambda_{m}^{\prime }/\left(\frac{1}{N_{m}}\sum_{k=1}^{N_{m}}{∥R_{k}∥}^{2}\right)$$

where $N_{m}$ is the total number of miRNAs and $\lambda_{m}^{\prime }$ is typically set to 1.

Similarly, the GIP kernel similarity between two diseases $d_{i}$ and $d_{j}$ is derived from their interaction profiles, represented as column vectors $C_{i}$ and $C_{j}$ from *A*. The disease similarity is calculated as follows:

(12) $$K_{d}\left(d_{i},d_{j}\right)=\exp\left(-\lambda_{d}{∥C_{i}-C_{j}∥}^{2}\right)$$

The bandwidth parameter $\lambda_{d}$ is set as follows:

(13) $$\lambda_{d}=\lambda_{d}^{\prime }/\left(\frac{1}{N_{d}}\sum_{k=1}^{N_{d}}{∥C_{k}∥}^{2}\right)$$

where $N_{d}$ denotes the total number of diseases and $\lambda_{d}^{\prime }$ is similarly assigned a value of 1.

#### 4.1.6. lncRNA-Based Similarity

Emerging evidence indicates that lncRNAs function as critical regulatory elements in diverse cellular mechanisms such as DNA methylation and post-transcriptional control, establishing meaningful connections between miRNA and disease pathways [64]. Building upon these functional linkages, MV-HAGCN employs lncRNA association patterns to quantify inter-molecule similarities.

The required interaction data were sourced from StarBase v2.0 [65] for raw sequences and from GATMDA [66] for pre-compiled miRNA–lncRNA and disease–lncRNA association matrices. We applied the edit distance algorithm [67] to these datasets to generate lncRNA-centric similarity matrices, producing $S_{\mathrm{lncRNA}}^{m}\in R^{N_{m}\times N_{m}}$ for miRNA relationships and $S_{\mathrm{lncRNA}}^{d}\in R^{N_{d}\times N_{d}}$ for disease relationships.

**Figure 11:**
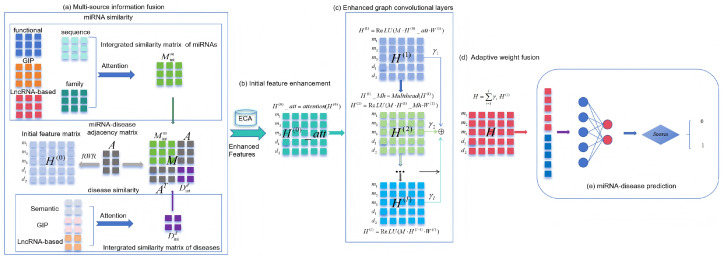
The overall architecture of MV-HAGCN. The framework consists of five key stages: (**a**) Multi-source information fusion: miRNA and disease similarities are integrated via attention, the RWR algorithm is then applied to the known miRNA–disease association matrix to generate initial node features; (**b**) Initial feature enhancement: the initial features are refined by an ECA module, which adaptively recalibrates feature channels to highlight informative dimensions; (**c**) Enhanced graph convolutional layers: two GCN layers with multi-head self-attention capture local and global dependencies; (**d**) Adaptive weight fusion: outputs from both layers are adaptively combined; (**e**) mirna-disease prediction: fused features are fed into an MLP to produce association scores.

The overall architecture of MV-HAGCN is illustrated in Figure 11. Its architecture can be categorized into five major components:

- Multi-source information fusion;
- Initial feature enhancement;
- Enhanced graph convolutional layers;
- Adaptive weight fusion;
- miRNA-disease prediction.

### 4.2. Multi-Source Information Fusion

MicroRNAs regulate biological processes through complex mechanisms that cannot be fully captured by any single similarity measure. Functional similarity reflects shared involvement in biological pathways [62]; sequence similarity indicates evolutionary conservation and potential target commonality [68]; family similarity captures structural and functional relatedness based on seed region conservation [69]; Gaussian kernel similarity (GIP) leverages known association patterns to infer functional proximity [70]; and lncRNA-based similarity bridges miRNA relationships through their shared interactions with lncRNAs [71]. Each perspective provides complementary information, and integrating them enables a more comprehensive and robust characterization of miRNA–miRNA relationships.

To obtain a comprehensive characterization of miRNA and disease relationships, we have acquired multiple similarity metrics: functional similarity (*FM*), Gaussian kernel similarity (*KM*), lncRNA-based similarity (*LM*) for miRNAs, sequence similarity (*SM*) and family similarity (*FAM*). These complementary measures are combined through a weighted fusion scheme to form an integrated miRNA similarity matrix:

(14) $$M_{int}^{m}=\alpha_{1}\mathrm{FM}+\alpha_{2}\mathrm{KM}+\alpha_{3}\mathrm{LM}+\alpha_{4}\mathrm{SM}+\alpha_{5}\mathrm{FAM}$$

where $\alpha_{1}$ through $\alpha_{5}$ represent the fusion coefficients that balance the contributions from different similarity sources.

Similarly, for diseases, we integrate semantic similarity (*SD*), Gaussian kernel similarity (*KD*), and lncRNA-based similarity (*LD*) into a unified disease similarity representation:

(15) $$M_{int}^{d}=\beta_{1}\mathrm{SD}+\beta_{2}\mathrm{KD}+\beta_{3}\mathrm{LD}$$

with $\beta_{1}$, $\beta_{2}$, and $\beta_{3}$ denoting the corresponding weighting parameters. The optimal values for all fusion coefficients will be explored in the experimental analysis.

These integrated similarity matrices are subsequently employed to construct a heterogeneous miRNA-disease association network $N_{heter}$, which serves as the foundation for the multi-layer graph convolutional architecture. Its adjacency matrix is defined as follows:

(16) $$M=\left[\begin{array}{cc} M_{int}^{m} & A\\ A^{T} & M_{int}^{d} \end{array}\right]$$

where $M_{int}^{m}\in R^{N_{m}\times N_{m}}$, $M_{int}^{d}\in R^{N_{d}\times N_{d}}$, and $A\in R^{N_{m}\times N_{d}}$, and $M\in R^{\left(N_{d}+N_{m}\right)\times \left(N_{d}+N_{m}\right)}$.

Finally, to initialize the features of miRNAs and diseases, MV-HAGCN takes advantage of the Random Walk with Restart (RWR) algorithm [72] in the adjacency matrix A, which serves as the baseline input. Compared with simpler initialization strategies such as degree normalization or Laplacian eigenvectors, RWR captures global topological information by simulating diffusion processes with a restart probability, enabling the model to generate smooth and context-aware initial node features that reflect the underlying network structure. The RWR procedure is formally defined as follows:

(17) $$D_{t}^{\left(k\right)}=\left(1-r\right)A_{N}D_{t}^{\left(k-1\right)}+rD_{t}^{\left(0\right)}$$

where *r* denotes the restart probability, *t* the iteration number, $D_{t}^{\left(0\right)}\in R^{(N_{d}+N_{m})\times 1}$ the initial vector for the *t*-th node, and $A_{n}$ the normalized adjacency matrix. Iterations proceed until $|D^{\left(k+1\right)}-D^{\left(k\right)}{|}_{F}\leq 10^{-6}$, where $D_{t}^{\left(k\right)}$ is the *k*-th iterative feature of node *t*. The resultant feature matrix $D^{K}$ thus contains all initial miRNA and disease features.

### 4.3. Initial Feature Enhancement

We apply an attention mechanism to node features before graph convolution. At the input layer, the Efficient Channel Attention (ECA) mechanism is employed to adaptively recalibrate the importance of each channel in the input features. The corresponding formulas are as follows:

(18) Attention=σ(Conv1D(GlobalAvgPool(X)))

(19) $$X_{\mathrm{Enhanced}}=X⊙\mathrm{Attention}$$

(20) $$H_{\mathrm{att}}^{\left(0\right)}=\mathrm{attention}\left(H^{\left(0\right)}\right)$$

where *X* denotes the input feature matrix, GlobalAvgPool represents the global average pooling operation, Conv1D is the 1D convolution operation, the sigmoid activation function σ normalizes weights to the [0, 1] range, Attention is the computed channel attention weight, ⊙ denotes element-wise multiplication (Hadamard product), $X_{\mathrm{Enhanced}}$ is the enhanced feature after attention weighting, $H^{\left(0\right)}$ is the initial node feature matrix obtained from the RWR algorithm, and $H_{\mathrm{att}}$ is the initial feature representation enhanced by the ECA attention mechanism.

### 4.4. Enhanced Graph Convolutional Layers

**Figure 12:**
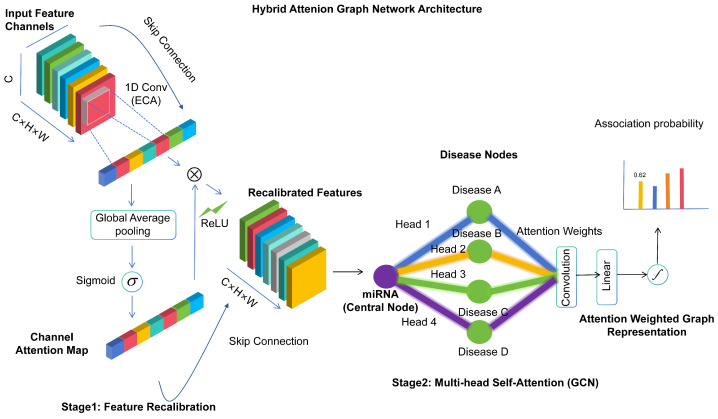
Detailed architecture of the hybrid attention graph network. The framework consists of two sequential stages. Stage 1 (Feature Recalibration): The Efficient Channel Attention (ECA) module first performs global average pooling to aggregate spatial information, followed by a 1D convolution that adaptively determines the coverage of local cross-channel interactions. The resulting attention weights are normalized via a sigmoid function and then multiplied element-wise with the input features, producing recalibrated feature representations. A skip connection is incorporated to preserve the original information. Stage 2 (Multi-Head Self-Attention Enhanced GCN): The recalibrated features are fed into a multi-head self-attention module, which captures global dependencies among nodes by computing attention scores across multiple representation subspaces. The attended features are then passed through a graph convolutional layer that aggregates information from neighboring nodes, effectively integrating local structural information with global contextual relationships. This dual-stage design enables the model to jointly optimize feature importance and topological dependencies for improved association prediction.

Within the graph convolutional layer, we integrate a multi-head self-attention mechanism to capture complex dependencies between nodes and adaptively adjust the importance of node features. This forms a dual attention mechanism that encompasses both in feature-wise and structural attention, significantly enhancing the feature representation capability.The detail is shown in Figure 12.

The first graph convolutional layer with attention-enhanced features is formulated as follows:

(21) $$H^{\left(1\right)}=\mathrm{ReLU}\left(M\cdot H_{\mathrm{att}}^{\left(0\right)}\cdot W^{\left(1\right)}\right)$$

where *M* is the adjacency matrix of the heterogeneous network, $H_{\mathrm{att}}^{\left(0\right)}$ is the initial feature enhanced by ECA attention, $W^{\left(1\right)}$ is the learnable weight matrix of the first graph convolutional layer, ReLU is the activation function that introduces nonlinearity, and $H^{\left(1\right)}$ is the output feature of the first graph convolutional layer, capturing meta-path information of length 1.

The multi-head self-attention mechanism applied to the first layer output is defined as follows:

(22) $$H_{\mathrm{mh}}^{\left(1\right)}=\mathrm{MultiHead}\left(H^{\left(1\right)}\right)$$

where MultiHead is the multi-head self-attention function (defined by Equations (25)–(27), $H_{\mathrm{mh}}^{\left(1\right)}$ is the feature processed by multi-head self-attention, capturing high-order dependencies between nodes.

The second graph convolutional layer with multi-head self-attention features is formulated as follows:

(23) $$H^{\left(2\right)}=\mathrm{ReLU}\left(M\cdot H_{\mathrm{mh}}^{\left(1\right)}\cdot W^{\left(2\right)}\right)$$

where $W^{\left(2\right)}$ denotes the trainable parameter matrix in the second graph convolutional layer, and $H^{\left(2\right)}$ corresponds to the output representation from this layer, which encodes meta-path information of length 2.

The general formulation for the (l+1)-th graph convolutional layer is given as follows:

(24) $$H^{\left(l+1\right)}=\mathrm{ReLU}\left(M\cdot H^{\left(l\right)}\cdot W^{\left(l+1\right)}\right)$$

where $H^{\left(l\right)}$ is the feature representation of the *l*-th layer, $W^{\left(l+1\right)}$ is the learnable weight matrix of the (l+1)-th layer, $H^{\left(l+1\right)}$ is the output feature of the (l+1)-th layer, capturing meta-path information of length l+1.

The multi-head self-attention mechanism is then constructed by concatenating all attention heads and applying a linear transformation:

(25) $${\mathrm{Head}}_{i}=\mathrm{Attention}\left(XW_{i}^{Q},XW_{i}^{K},XW_{i}^{V}\right)$$

(26) $$\mathrm{MultiHead}\left(X\right)=\mathrm{Concat}\left({\mathrm{Head}}_{1},\ldots ,{\mathrm{Head}}_{h}\right)W^{O}$$

where the core attention function is defined as follows:

(27) $$\mathrm{Attention}\left(Q,K,V\right)=\mathrm{softmax}\left(\frac{QK^{T}}{\sqrt{d_{k}}}\right)V$$

### 4.5. Adaptive Weight Fusion

Multi-layer graph convolutional networks capture complementary structural information at different propagation depths: shallower layers preserve local neighborhood information, while deeper layers encode higher-order topological patterns. However, simply concatenating or averaging features from different layers may not optimally leverage their complementary strengths [73]. To address this, we introduce an adaptive weight fusion mechanism that learns to assign importance coefficients to each layer’s output based on their contributions to the final prediction. This approach enables the model to dynamically balance local and global structural information, mitigating the risk of over-smoothing while preserving fine-grained node features. The outputs from each layer are fused through adaptive weights:

(28) $$H=\gamma_{1}H^{\left(1\right)}\;+\gamma_{2}H^{\left(2\right)}\;$$

where *H* denotes the integrated output from the multi-layer GCN, representing the unified feature embedding for both miRNAs and diseases, $H^{\left(1\right)}$ and $H^{\left(2\right)}$ are the outputs of the first and second GCN layers respectively, and $\gamma_{1}$ through $\gamma_{2}$ are learnable parameters that adaptively adjust the contribution of each representation during training.

### 4.6. miRNA-Disease Prediction

After obtaining the unified feature representations through adaptive fusion, we employ a multi-layer perceptron (MLP) to map these embeddings to association scores. MLPs are capable of learning complex non-linear decision boundaries and have been widely adopted in miRNA-disease association prediction tasks due to their flexibility and strong representation power [74]. The integrated features are passed to a multi-layer perceptron for the final prediction:

(29) $$\hat{Y}=\mathrm{MLP}\left(H\right)$$

The model is trained by minimizing the binary cross-entropy loss:

(30) $$L=-\frac{1}{N}\sum_{(i,j)\in D}\left[y_{ij}\log\left({\hat{y}}_{ij}\right)+\left(1-y_{ij}\right)\log\left(1-{\hat{y}}_{ij}\right)\right]$$

where *D* denotes the training set of miRNA-disease pairs, and $y_{ij}\in \left\{0,1\right\}$ represents the true association label.

### 4.7. Time Complexity Analysis

We analyze the computational complexity of MV-HAGCN to assess its scalability. Let N=m+n denote the total number of nodes (where *m* and *n* are the numbers of miRNAs and diseases, respectively), and let *d* be the feature dimension. The overall complexity is dominated by three main components:

- Multi-source fusion: Constructing the integrated similarity matrices requires $O(m^{2}+n^{2})$ operations, corresponding to pairwise comparisons within each node type.
- Hierarchical attention and graph convolution: For each of the *L* graph convolutional layers, the multi-head self-attention mechanism and adjacency matrix multiplication contribute $O(N^{2}\cdot d)$ per layer. The total cost across all layers is therefore $O(L\cdot N^{2}\cdot d)$.
- Overall complexity: Combining the above, the dominant term is $O(L\cdot N^{2}\cdot d)$. With fixed *L* and *d* (in our experiments, L=2, d=256), this simplifies to $O({\left(m+n\right)}^{2})$, i.e., quadratic in the total number of nodes.

This quadratic complexity is acceptable for current miRNA–disease datasets. For instance, HMDD v3.2 contains approximately 850 miRNAs and 600 diseases, yielding N≈1450; training MV-HAGCN for 2000 epochs on this dataset required approximately 1291 s on an NVIDIA GeForce RTX 3050 Ti Laptop GPU (NVIDIA Corporation, Santa Clara, CA, USA), confirming its practical feasibility. A comprehensive comparison of training times for MV-HAGCN and all baseline methods is provided in Supplementary Table S8. However, scaling to much larger biomedical networks (e.g., with tens of thousands of nodes) may require future optimizations, such as graph sampling or sparse attention mechanisms. This analysis confirms that MV-HAGCN achieves a favorable balance between expressiveness and computational feasibility for practical applications.

### 4.8. Experimental Setup

All experiments were conducted on a workstation equipped with an NVIDIA GeForce RTX 3050 Ti Laptop GPU (NVIDIA Corporation, Santa Clara, CA, USA). The model was implemented using Python 3.8 with the following key libraries:PyTorch 1.9.1+cu111, NumPy 1.19.2, SciPy 1.5.4, Pandas 0.25.0. These details are provided to ensure the reproducibility of our experimental results.

## 5. Conclusions

This study proposes an innovative multi-view hybrid attention graph convolutional network for predicting miRNA-disease associations. By constructing a multi-level feature fusion architecture, the model effectively integrates complementary biological information—including miRNA functional similarity, sequence similarity, and disease semantic similarity—into a comprehensive heterogeneous network. The incorporation of an Efficient Channel Attention mechanism and multi-head self-attention within the graph convolutional layers enables enhanced feature representation and dual attention to both local and global node features. Adaptive fusion of multi-source information is further achieved through dynamic weight learning.

Extensive experimental results confirm that our framework consistently outperforms existing state-of-the-art methods across multiple evaluation metrics. Ablation studies further validate the indispensable contributions of the multi-source fusion module and the multi-head self-attention graph convolutions. Case studies also illustrate the practical utility of the model in identifying potential miRNA candidates for major diseases.

## Data Availability

The data and codes supporting this work are available at https://github.com/konglinXing/MV-HAGCN-master (accessed on 12 February 2026).

## Supplementary Materials

The following supporting information can be downloaded at: https://www.mdpi.com/article/10.3390/ijms27083533/s1.
