# Supplementary material for: MV-HAGCN: Prediction of miRNA-Disease Association Based on Multi-View Hybrid Attention Graph Convolutional Network
Source: Int J Mol Sci. 2026 Apr 15;27(8):3533. doi: 10.3390/ijms27083533 (PMC13115663; doi:10.3390/ijms27083533)
Supplement: Supplementary file 1 [file ijms-27-03533-s001.zip › ijms-4174507-supplementary.pdf]

Supplementary Materials

KongLin Xing

March 2026

Note: The symbol “+” indicates that the miRNA–disease association has been confirmed in existing databases; “-” (not present in these tables) would indicate a novel prediction without experimental confirmation.

Table S1: Top 50 breast neoplasms-related miRNAs predicted by MV-HAGCN with prediction scores

Rank	miRNA	Evidence	Score	Rank(Cont.)	miRNA	Evidence	Score
1	hsa-mir-301a	+	0.689	26	hsa-let-7i	+	0.537
2	hsa-mir-301b	+	0.682	27	hsa-mir-375	+	0.530
3	hsa-mir-132	+	0.676	28	hsa-mir-7	+	0.524
4	hsa-mir-373	+	0.669	29	hsa-mir-107	+	0.517
5	hsa-let-7a	+	0.662	30	hsa-mir-335	+	0.510
6	hsa-mir-30b	+	0.656	31	hsa-mir-223	+	0.504
7	hsa-let-7f	+	0.649	32	hsa-mir-302c	+	0.497
8	hsa-let-7b	+	0.642	33	hsa-mir-125a	+	0.490
9	hsa-mir-18a	+	0.636	34	hsa-mir-140	+	0.484
10	hsa-let-7g	+	0.629	35	hsa-mir-29a	+	0.477
11	hsa-let-7d	+	0.622	36	hsa-mir-200a	+	0.470
12	hsa-let-7c	+	0.616	37	hsa-mir-19a	+	0.464
13	hsa-mir-17	+	0.609	38	hsa-mir-192	+	0.457
14	hsa-mir-141	+	0.602	39	hsa-mir-302d	+	0.450
15	hsa-mir-101	+	0.596	40	hsa-mir-203	+	0.444
16	hsa-mir-19b	+	0.589	41	hsa-mir-15a	+	0.437
17	hsa-mir-23b	+	0.582	42	hsa-mir-214	+	0.430
18	hsa-mir-30c	+	0.576	43	hsa-mir-302a	+	0.424
19	hsa-mir-181b	+	0.569	44	hsa-let-7e	+	0.417
20	hsa-mir-376c	+	0.562	45	hsa-mir-18b	+	0.410
21	hsa-mir-29c	+	0.556	46	hsa-mir-520c	+	0.404
22	hsa-mir-125b	+	0.549	47	hsa-mir-20a	+	0.397
23	hsa-mir-194	+	0.542	48	hsa-mir-205	+	0.390
24	hsa-mir-196a	+	0.536	49	hsa-mir-200c	+	0.384
25	hsa-mir-135b	+	0.529	50	hsa-mir-299	+	0.377

Table S2: Top 50 lung neoplasms-related miRNAs predicted by MV-HAGCN with prediction scores

Rank	miRNA	Evidence	Score	Rank(Cont.)	miRNA	Evidence	Score
1	hsa-mir-1	+	0.683	26	hsa-mir-18b	+	0.519
2	hsa-mir-133a	+	0.676	27	hsa-mir-494	+	0.512
3	hsa-mir-206	+	0.670	28	hsa-mir-34c	+	0.506
4	hsa-mir-32	+	0.663	29	hsa-mir-497	+	0.499
5	hsa-mir-133b	+	0.657	30	hsa-mir-18a	+	0.492
6	hsa-mir-132	+	0.650	31	hsa-mir-301a	+	0.486
7	hsa-mir-424	+	0.644	32	hsa-mir-142	+	0.479
8	hsa-mir-30e	+	0.637	33	hsa-mir-23b	+	0.473
9	hsa-mir-205	+	0.630	34	hsa-mir-24	+	0.466
10	hsa-let-7d	+	0.624	35	hsa-mir-93	+	0.460
11	hsa-let-7e	+	0.617	36	hsa-mir-125b	+	0.453
12	hsa-mir-125a	+	0.611	37	hsa-mir-136	+	0.446
13	hsa-let-7f	+	0.604	38	hsa-mir-335	+	0.440
14	hsa-let-7b	+	0.598	39	hsa-mir-30c	+	0.433
15	hsa-let-7g	+	0.591	40	hsa-mir-100	+	0.427
16	hsa-mir-196a	+	0.584	41	hsa-mir-375	+	0.420
17	hsa-let-7c	+	0.578	42	hsa-mir-34a	+	0.414
18	hsa-mir-499a	+	0.571	43	hsa-mir-212	+	0.407
19	hsa-mir-101	+	0.565	44	hsa-mir-19a	+	0.400
20	hsa-mir-192	+	0.558	45	hsa-mir-30b	+	0.394
21	hsa-mir-1297	+	0.552	46	hsa-let-7i	+	0.387
22	hsa-mir-98	+	0.545	47	hsa-mir-181a	+	0.381
23	hsa-let-7a	+	0.538	48	hsa-mir-17	+	0.374
24	hsa-mir-7	+	0.532	49	hsa-mir-137	+	0.368
25	hsa-mir-140	+	0.525	50	hsa-mir-145	+	0.361

Table S3: Top 50 pancreatic neoplasms-related miRNAs predicted by MV-HAGCN with prediction scores

Rank	miRNA	Evidence	Score	Rank(Cont.)	miRNA	Evidence	Score
1	hsa-let-7c	+	0.679	26	hsa-mir-15a	+	0.519
2	hsa-let-7f	+	0.673	27	hsa-mir-100	+	0.512
3	hsa-let-7i	+	0.666	28	hsa-mir-101	+	0.506
4	hsa-let-7g	+	0.660	29	hsa-mir-106a	+	0.500
5	hsa-mir-132	+	0.653	30	hsa-mir-196a	+	0.493
6	hsa-let-7d	+	0.647	31	hsa-mir-17	+	0.487
7	hsa-let-7b	+	0.641	32	hsa-mir-32	+	0.480
8	hsa-mir-148a	+	0.634	33	hsa-mir-18a	+	0.474
9	hsa-mir-338	+	0.628	34	hsa-mir-23b	+	0.467
10	hsa-mir-221	+	0.621	35	hsa-mir-181b	+	0.461
11	hsa-mir-200a	+	0.615	36	hsa-mir-133b	+	0.455
12	hsa-let-7a	+	0.608	37	hsa-mir-145	+	0.448
13	hsa-mir-222	+	0.602	38	hsa-mir-135b	+	0.442
14	hsa-mir-192	+	0.596	39	hsa-mir-10a	+	0.435
15	hsa-mir-194	+	0.589	40	hsa-mir-130a	+	0.429
16	hsa-mir-15b	+	0.583	41	hsa-mir-125b	+	0.423
17	hsa-mir-34c	+	0.576	42	hsa-mir-92a	+	0.416
18	hsa-mir-214	+	0.570	43	hsa-mir-31	+	0.410
19	hsa-mir-223	+	0.564	44	hsa-mir-146b	+	0.403
20	hsa-mir-375	+	0.557	45	hsa-mir-25	+	0.397
21	hsa-mir-107	+	0.551	46	hsa-mir-34a	+	0.391
22	hsa-mir-146a	+	0.544	47	hsa-mir-20a	+	0.384
23	hsa-mir-200b	+	0.538	48	hsa-mir-29b	+	0.378
24	hsa-mir-126	+	0.532	49	hsa-mir-218	+	0.371
25	hsa-let-7e	+	0.525	50	hsa-mir-29a	+	0.365

Table S4: Top 50 esophageal neoplasms-related miRNAs predicted by MV-HAGCN with prediction scores

Rank	miRNA	Evidence	Score	Rank(Cont.)	miRNA	Evidence	Score
1	hsa-mir-375	+	0.685	26	hsa-mir-221	+	0.519
2	hsa-mir-143	+	0.678	27	hsa-mir-192	+	0.513
3	hsa-mir-31	+	0.672	28	hsa-mir-19a	+	0.506
4	hsa-mir-145	+	0.665	29	hsa-mir-196a	+	0.499
5	hsa-mir-223	+	0.658	30	hsa-mir-203	+	0.493
6	hsa-mir-302a	+	0.652	31	hsa-mir-548d	+	0.486
7	hsa-mir-205	+	0.645	32	hsa-mir-302f	+	0.479
8	hsa-mir-126	+	0.639	33	hsa-mir-214	+	0.473
9	hsa-let-7a	+	0.632	34	hsa-mir-451a	+	0.466
10	hsa-mir-34a	+	0.625	35	hsa-mir-101	+	0.459
11	hsa-mir-28	+	0.619	36	hsa-mir-200a	+	0.453
12	hsa-mir-146a	+	0.612	37	hsa-mir-15a	+	0.446
13	hsa-let-7c	+	0.605	38	hsa-mir-34c	+	0.440
14	hsa-mir-342	+	0.599	39	hsa-mir-206	+	0.433
15	hsa-let-7b	+	0.592	40	hsa-mir-32	+	0.426
16	hsa-mir-148a	+	0.586	41	hsa-mir-486	+	0.420
17	hsa-mir-155	+	0.579	42	hsa-let-7i	+	0.413
18	hsa-mir-133a	+	0.572	43	hsa-mir-720	+	0.406
19	hsa-mir-130a	+	0.566	44	hsa-mir-1	+	0.400
20	hsa-mir-23b	+	0.559	45	hsa-mir-34b	+	0.393
21	hsa-mir-20a	+	0.552	46	hsa-mir-17	+	0.387
22	hsa-mir-455	+	0.546	47	hsa-mir-574	+	0.380
23	hsa-mir-125b	+	0.539	48	hsa-mir-107	+	0.373
24	hsa-mir-98	+	0.532	49	hsa-mir-22	+	0.367
25	hsa-mir-132	+	0.526	50	hsa-mir-222	+	0.360

Table S5: Top 50 lymphoma-related miRNAs predicted by MV-HAGCN with prediction scores

Rank	miRNA	Evidence	Score	Rank(Cont.)	miRNA	Evidence	Score
1	hsa-mir-19a	+	0.680	26	hsa-mir-222	+	0.514
2	hsa-mir-19b	+	0.673	27	hsa-mir-34c	+	0.508
3	hsa-mir-17	+	0.667	28	hsa-mir-424	+	0.501
4	hsa-mir-155	+	0.660	29	hsa-mir-29b	+	0.494
5	hsa-mir-18a	+	0.653	30	hsa-mir-145	+	0.488
6	hsa-mir-20a	+	0.647	31	hsa-mir-106b	+	0.481
7	hsa-mir-146a	+	0.640	32	hsa-mir-7	+	0.474
8	hsa-mir-372	+	0.634	33	hsa-mir-223	+	0.468
9	hsa-mir-28	+	0.627	34	hsa-mir-126	+	0.461
10	hsa-mir-15b	+	0.620	35	hsa-mir-29c	+	0.454
11	hsa-mir-92a	+	0.614	36	hsa-let-7f	+	0.448
12	hsa-mir-29a	+	0.607	37	hsa-mir-142	+	0.441
13	hsa-mir-23b	+	0.600	38	hsa-mir-200b	+	0.435
14	hsa-mir-16	+	0.594	39	hsa-mir-214	+	0.428
15	hsa-mir-200a	+	0.587	40	hsa-let-7g	+	0.421
16	hsa-mir-125b	+	0.581	41	hsa-mir-494	+	0.415
17	hsa-mir-221	+	0.574	42	hsa-mir-132	+	0.408
18	hsa-let-7c	+	0.567	43	hsa-mir-34a	+	0.401
19	hsa-let-7b	+	0.561	44	hsa-mir-373	+	0.395
20	hsa-mir-133a	+	0.554	45	hsa-mir-195	+	0.388
21	hsa-mir-196a	+	0.547	46	hsa-mir-150	+	0.382
22	hsa-let-7a	+	0.541	47	hsa-mir-192	+	0.375
23	hsa-mir-181a	+	0.534	48	hsa-mir-205	+	0.368
24	hsa-mir-15a	+	0.527	49	hsa-mir-133b	+	0.362
25	hsa-let-7d	+	0.521	50	hsa-mir-342	+	0.355

Table S6: Performance comparison of different methods on HMDD v3.2 dataset (mean ± std).

**Model**	**AUC**	**AUPR**	**ACC**	**F1-Score**	**Recall**
AGAEAMD	0.9037 ± 0.0167	0.9071 ± 0.0140	0.8501 ± 0.0126	0.8498 ± 0.0143	0.8534 ± 0.0145
AMHMDA	0.9419 ± 0.0067	0.9366 ± 0.0162	0.8667 ± 0.0138	0.8643 ± 0.0123	0.8733 ± 0.0141
DGAMDA	0.9629 ± 0.0034	0.9588 ± 0.0044	0.8849 ± 0.0024	0.8929 ± 0.0041	0.8769 ± 0.0039
ESGC-MDA	0.9503 ± 0.0026	0.9503 ± 0.0031	0.8812 ± 0.0135	0.8863 ± 0.0139	0.8747 ± 0.0136
MHCLMDA	0.9340 ± 0.0066	0.9392 ± 0.0063	0.8749 ± 0.0067	0.8876 ± 0.0056	0.8801 ± 0.0055
MINIMDA	0.9406 ± 0.0087	0.9277 ± 0.0160	0.8481 ± 0.0134	0.8541 ± 0.0121	0.8761 ± 0.0146
MV-HAGCN	$\mathbf{0.9882 \pm 0.0017}$	$\mathbf{0.9897 \pm 0.0015}$	$\mathbf{0.9502 \pm 0.0026}$	$\mathbf{0.9497 \pm 0.0035}$	$\mathbf{0.9412 \pm 0.0043}$

Table S7: Quantitative evaluation of case study results for five diseases

**Disease**	**Known associations**	**Precision@50**	**Recall@50**	**Hit Rate@50**
Breast neoplasms	5806	100% (50/50)	0.86% (50/5806)	100%
Lung neoplasms	2818	100% (50/50)	1.77% (50/2818)	100%
Pancreatic neoplasms	691	100% (50/50)	7.23% (50/691)	100%
Esophageal neoplasms	440	100% (50/50)	11.36% (50/440)	100%
Lymphoma	236	100% (50/50)	21.18% (50/236)	100%

Note: Known associations refer to the total number of experimentally validated miRNA–disease associations recorded in HMDD v4.0 and dbDEMC for each disease. Precision@50 is calculated as the proportion of top 50 predictions confirmed in these databases. Recall@50 is the percentage of known associations recovered within the top 50 predictions. Hit Rate@50 indicates whether at least one valid association was found in the top 50 (100% for all diseases).

Table S8: Training time comparison of MV-HAGCN and baseline methods

**Model**	**Epochs**	**Total training time (s)**	**Average time per epoch (s)**
AGAEMD	5000	2200	0.440
AMHMDA	1200	1452	1.210
DGAMDA	1000	1505	1.505
ESGC-MDA	2000	1180	0.590
MHCLMDA	1350	440	0.325
MINIMDA	1000	1789	1.789
MAGMDA	3600	1701	0.472
MV-HAGCN	2000	1291	0.646

Note: All models were run with their original parameters on an NVIDIA GeForce RTX 3050 Ti GPU. Total training time is reported in seconds, and the average time per epoch is calculated as total time divided by the number of training epochs for each respective model.

Table S9: Prospective validation via time-course simulation using sequential HMDD versions

**Disease**	**Training data**	**Top-k predictions**	**Novel validations**	**Success rate**
Breast neoplasms	HMDD v2.0	50	4	8%

Note: Novel validations refer to associations that were absent in HMDD v2.0 (the training set) but first appeared in HMDD v3.2 or v4.0. The model was trained exclusively on HMDD v2.0 and then used to predict top-50 miRNAs for breast neoplasms. Success rate is the proportion of top-50 predictions that were later confirmed as novel associations in the updated databases.
